# Supplementary material for: Endogenous Extracellular Matrix Regulates the Response of Osteosarcoma 3D Spheroids to Doxorubicin
Source: Cancers (Basel). 2023 Feb 14;15(4):1221. doi: 10.3390/cancers15041221 (PMC9954237; doi:10.3390/cancers15041221)
Supplement: Supplementary file 1 [file cancers-15-01221-s001.zip › cancers-2202997-supplementary.pdf]

Supplementary Material

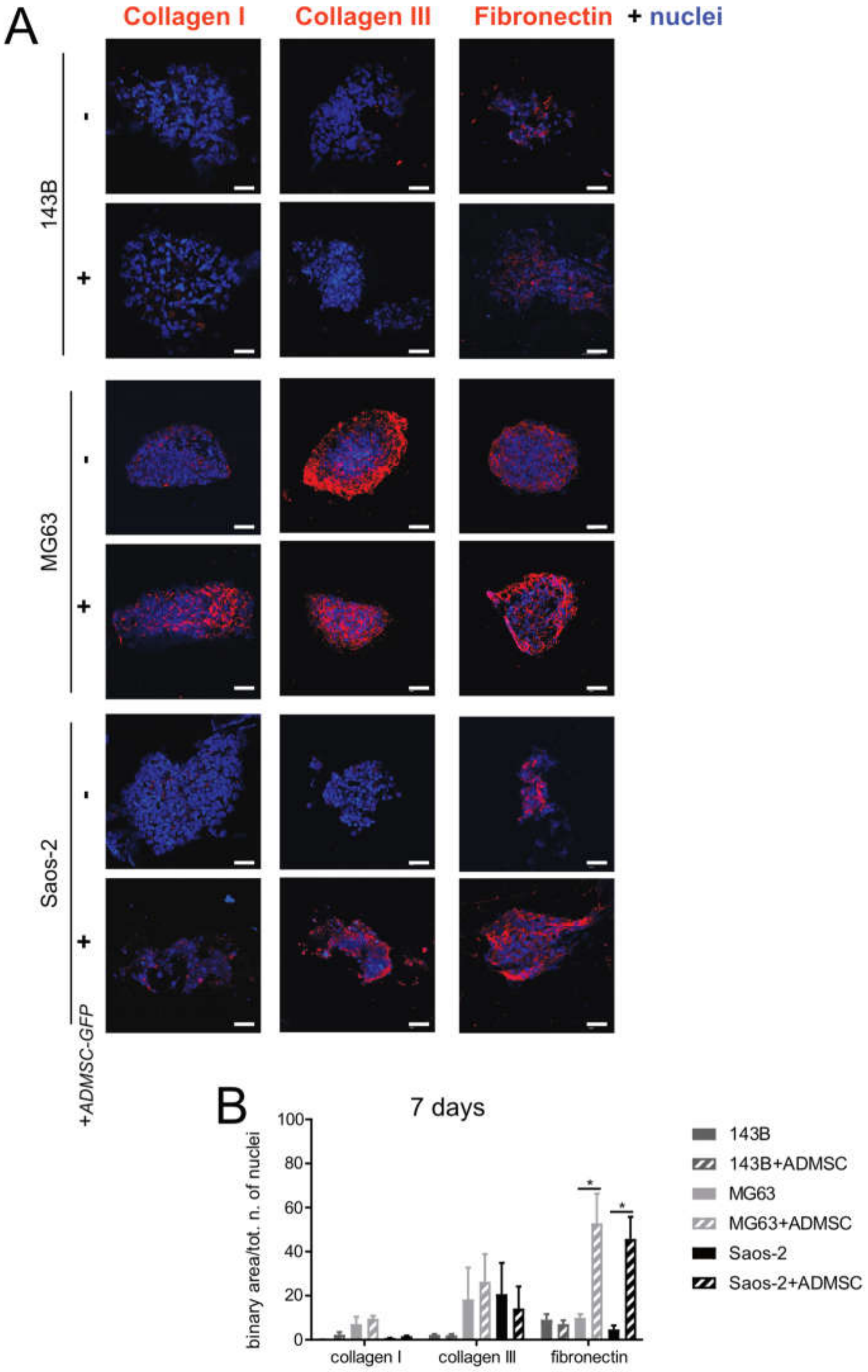

Figure S1. Day 7 expression of ECM proteins. A) Representative confocal images of immunofluorescence of OS spheroids from 3 different cell lines cultured as homotypic (OS cells alone), or as heterotypic mixed with mesenchymal stromal cells (OS/ADMSC-GFP spheroids), grown for 7 days. The spheroids were included in OCT, sliced and stained with specific antibodies for collagen Type I, collagen Type III and fibronectin (red) and counterstained with Hoechst (blue). (scale bar 50 µm); B) Quantification of IF shown in A, expressed as the total area occupied by ECM protein, and divided for the total number of nuclei. Data presented as mean ± SEM of n=10 different fields from 3 independent experiments (unpaired two-tailed Mann-Whitney test; ***$p<0.001$; **$p< 0.01$; *$p<0.05$).

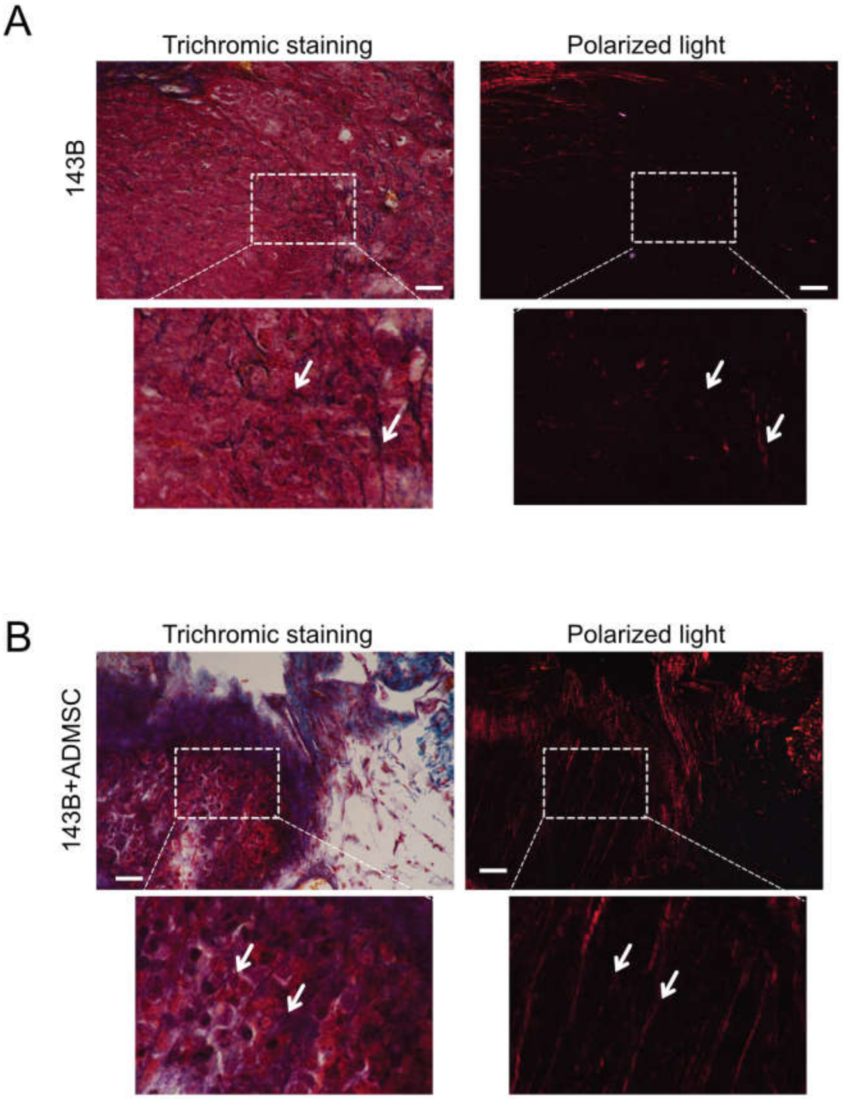

Figure S2. Collagen expression in intratibial OS tumors. A and B) Trichromic staining (left) and polarized light (right) of orthotopic intratibial OS 143B tumors without ADMSC (A) or with ADMSC in a 3:1 ratio (B) were included in paraffin and sliced at 7 µm. Collagen appears in blue in the trichromic staining and in red in polarized light images. Scale bar 100 µm. Arrows indicate positive staining.

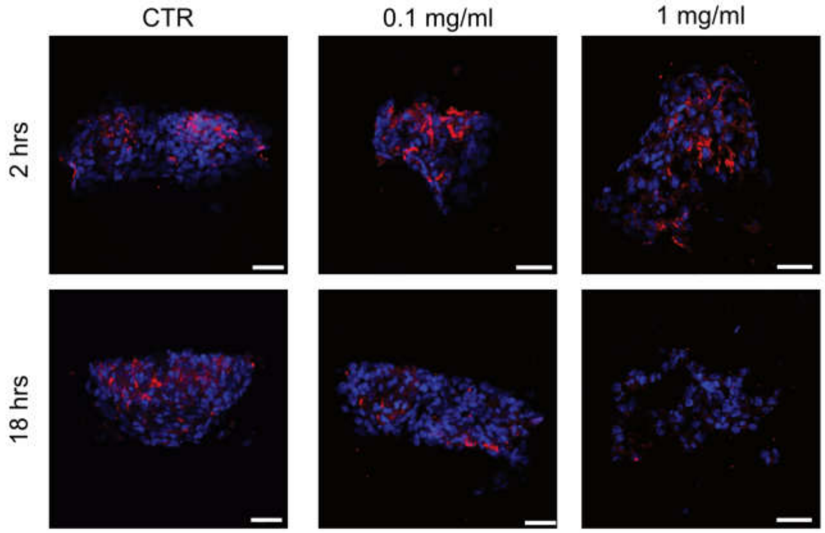

Figure S3. Collagenase treatment does not affect collagen Type III staining. Spheroids from MG63/ADMSC-GFP grown for 14 days, treated with collagenase IA as indicated, included in OCT, sliced at 5 μm and stained for collagen Type III, counterstained with Hoechst (blue). Scale bar 50 μm.
